# Medical students’ knowledge, attitudes, and practices regarding artificial intelligence: A cross-sectional study

**DOI:** 10.1371/journal.pone.0350001

**Published:** 2026-10-27

**Authors:** Ahmed A. Mosa, Ibrahim A. Naqid, Aram A. Younus, Kavi A. Khorsheed, Nora Abdi Mahmoud, Araz Ali Younus, Hevi Sleman M. Ali, Zana Yousif Sulaiman

**Affiliations:** 1 Department of Medical Education, College of Medicine, University of Zakho, Zakho, Kurdistan Region, Iraq; 2 Department of Biomedical Sciences, College of Medicine, University of Zakho, Zakho, Kurdistan Region, Iraq; 3 College of Medicine, University of Duhok, Duhok, Kurdistan Region, Iraq; Universiti Monash Malaysia: Monash University Malaysia, MALAYSIA

## Abstract

**Introduction and objectives:**

Artificial intelligence (AI) is a branch of computer science capable of mimicking human cognitive functions. The integration of AI has significantly transformed healthcare and medical education. In medical education, AI has played a crucial role in improving students’ educational experiences. However, evidence from low- and middle-income countries (LMICs) such as Iraq remains limited. Therefore, this study aimed to assess medical students’ knowledge, attitudes, and practices (KAP) toward AI in the Kurdistan Region of Iraq.

**Materials and methods:**

This cross-sectional study was carried out among medical students in the Kurdistan Region of Iraq, between 3^rd^ November 2025 and 20^th^ January 2026. A structured, self-administered questionnaire was delivered to students either by paper and pen or digitally using the Google Form platform. A total of 575 medical students were recruited. The final piloted questionnaire consisted of 39 items and was divided into five distinct sections: basic demographics, knowledge, attitudes, practices, and perceived barriers,

**Results:**

The mean age of the study participants was 21.31 ± 1.7 SD years, and the representation between both genders was approximately equal. Additionally, only one-fifth (19.7%) of the students had attended a workshop about AI in healthcare and medical education. The internet/social media was the most common information source utilized by the students (77.7%). Moreover, AI was most frequently used for homework/assignments (56.5%), and the most significant barrier limiting AI use was fear of over-reliance (53.2%). Female students had significantly lower odds of having better knowledge (adjusted odds ratio (aOR): 0.34; 95% confidence interval (CI): 0.22-0.51; p<0.001), and positive attitudes (aOR: 0.46; 95% CI: 0.28-0.77; p=0.003) compared to male counterparts. Additionally, students from Duhok Medical College had significantly lower odds of having positive attitudes (aOR: 0.52; 95% CI: 0.30-0.88; p=0.014).

**Conclusions:**

The present study revealed suboptimal levels of KAP regarding AI among the surveyed medical students. Integrating structured AI education into medical school curricula may strengthen students’ understanding and competencies in AI, thereby resulting in better preparedness for AI application in future practice.

## Introduction

Artificial intelligence (AI) is a branch of computer science that utilizes algorithms and models capable of mimicking human cognitive functions, including learning, reasoning, planning, and problem-solving [1]. AI encompasses machine learning, deep learning, representation learning, and natural language processing. In recent years, AI has been recognized as a key driver of the Fourth Industrial Revolution, fundamentally transforming work organization today [2]. Its applications have extended beyond computer science into diverse sectors including statistics, linguistics, psychology, philosophy, and medicine [3].

In healthcare, AI plays a vital and transformative role, beyond the optimization of administrative tasks such as patient check-ins, digitalization of medical records, and follow-up of patients [4]. AI has reduced workload, significantly enhanced diagnostic accuracy, strengthened treatment planning, and resulted in better disease prognosis across multiple medical specialties [5,6]. Furthermore, in medical education, AI has played a critical role in improving students’ educational experiences [7]. AI has facilitated virtual teaching and training for health sciences students, thereby supporting medical education and improving educational experiences [8,9]. Additionally, evidence suggests that AI has the potential to enhance medical students’ clinical reasoning, clinical competence, and knowledge acquisition by up to (46%) [10]. Despite these educational benefits, concerns regarding privacy and data security remain among the most prominent ethical challenges surrounding the implementation of AI and large language models (LLMs) in medical education [11].

Globally, the integration of AI into healthcare and medical education has significantly transformed the way that these professions function today. However, in the Kurdistan Region of Iraq, systematic endeavors for integration remain scarce. The adoption and integration of AI in our region face several challenges, including limited institutional infrastructure, absence of standardized curriculum, a shortage of trained professionals, and inadequate technological resources [12,13]. Collectively, these barriers hinder the effective implementation of AI in our region; this contributes to gaps in practical knowledge and competencies in AI among health professions students and medical students. Consequently, many students engage in informal or self-directed learning to acquire AI-related knowledge and skills [14].

It is becoming increasingly evident that AI will play an increasingly important role in shaping the future of healthcare and medical education. Consequently, understanding gaps in AI knowledge and readiness among medical students is essential, particularly in low- and middle-income countries (LMICs), including the Kurdistan Region of Iraq, where evidence on AI preparedness remains scarce. To address this gap, the primary objectives of the present study were to assess medical students’ knowledge, attitudes, and practices (KAP) toward AI in the Kurdistan Region of Iraq and to identify key factors associated with better KAP. A secondary objective was to explore barriers associated with AI usage among medical students. The findings of the study may guide the development of tailored programs for preparing competent graduates capable of utilizing AI methods to improve education and the health system.

## Materials and methods

### Study design and participants

This cross-sectional study was carried out among undergraduate medical students enrolled in Zakho Medical College and Duhok Medical College in the Kurdistan Region of Iraq. Data were collected from 3^rd^ November, 2025–20^th^ January, 2026. A convenience sampling method was employed to recruit participants. All eligible medical students were invited to voluntarily complete a structured, self-administered questionnaire utilized for this study. To increase response rate and ensure adequate representation across different academic years, both a paper-and-pen method and an online questionnaire using the Google Forms platform were employed. The online questionnaire was delivered to students using stage specific social media groups. However, to prevent duplicate responses, each academic stage was surveyed using only one data collection method. A total of 575 medical students were recruited in this study. The study design and methods were developed in alignment with the Standards for Strengthening the Reporting of Observational Studies in Epidemiology (STROBE) criteria.

About 1550 medical students were enrolled in these colleges. The required sample size for this targeted population, calculated at a (99%) confidence interval, a (5%) margin of error, and a response distribution of (50%) using an online sample size calculator (https://www.calculator.net/sample-size-calculator.html), was estimated to be 466 students. The recruited sample size is sufficient to effectively represent the targeted population. Fig 1 illustrates the participant recruitment flowchart.

**Fig 1:**
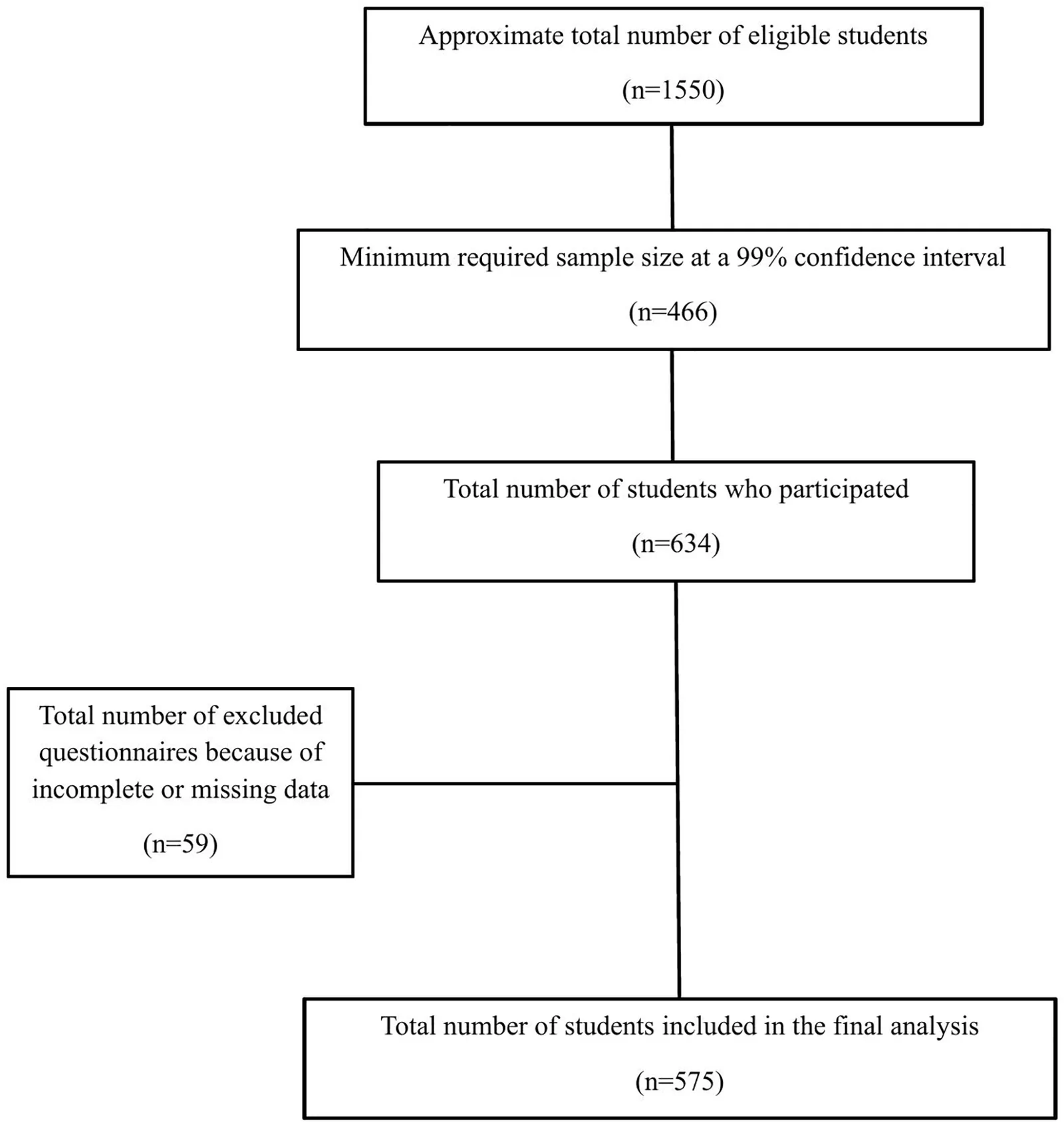
Participants recruitment flowchart.

### Instrument development

The questionnaire used in this study was developed based on an extensive review of the relevant literature [3,6,13,15]. Then, items from previous high-quality published and validated studies were adopted to explore medical students’ KAP toward AI. Furthermore, the adopted questionnaire was evaluated by two experts in the field of medical education and teaching and a research methodologist for clarity and comprehensiveness. Additionally, these experts reviewed the questionnaire for face and content validity as well as cross-cultural appropriateness. Content validation was carried out by two independent experts, who evaluated each item using a 4-point relevance scale, where 1 indicated non-relevant and 4 indicated highly relevant. The item-level content validity index (I-CVI) and the scale-level content validity/average method (S-CVI/Ave) were calculated from the experts’ ratings. For these calculations, ratings of 3 or 4 were recoded as 1 (relevant), whereas ratings of 1 or 2 were recoded as 0 (not relevant). All questionnaire items achieved an I-CVI of 1, except for one item in the knowledge domain, which had an I-CVI of 0.50. To resolve this disagreement, the item was further evaluated by a third expert, who recommended its exclusion. Following removal of this item, the S-CVI/Ave reached 1.00 for all study domains.

After finalization of the initial questionnaire draft, these experts trained six members of the research team before data collection to explain for them the study objectives, questionnaire items, and ethical considerations and to ensure standard and accurate administration of the questionnaire. A pilot test was conducted on 25 medical students prior to main data collection to check for clarity, cognitive understanding, and time required for completion. Following pilot testing, all questionnaire sections demonstrated strong clarity and comprehension, except the knowledge section of the questionnaire. Therefore, the expert team re-evaluated this section, and several refinements were made based on team consensus. The response scale was changed from “yes” and “no” to a 5-point Likert scale, and 4 new items were added. Later, this section was re-piloted on 15 students, and satisfactory results were obtained. Neither pilot testing samples were included in the final analysis.

### Study tool

The final piloted questionnaire consisted of 39 items and was divided into five distinct sections;

#### Section I – Basic demographic characteristics.

This section comprised 8 items and was designed to collect basic demographic and academic characteristics of the participants, including age, gender, college, stage, and cumulative academic average >70%. Furthermore, this section included items on students’ participation in AI workshops or seminars, as well as whether any initiatives were taken by their college to incorporate AI into education. Finally, this section was concluded by inquiring about the main sources of AI information among students.

#### Section II – Knowledge.

The second section consisted of 11 questions which were designed to explore students’ knowledge and awareness toward AI. The first four items evaluated students’ general knowledge about AI and its subtypes. The following three questions explored key limitations of AI and principal mechanisms through which AI systems operate. The final four items were related to students’ awareness about current applications of AI in medical fields, including radiology, pathology, and cardiology. The Cronbach’s alpha for the knowledge section was 0.79, which indicates satisfactory internal consistency of this section.

#### Section III – Attitude.

This section was designed to demonstrate participants’ attitudes toward AI in healthcare and medical education. It comprised 12 statements covering various aspects of attitudes. The first five items were related to educational use of AI and its potential influences on curriculum and assessment methods. The next five items explored students’ perceptions regarding the impact of AI on their career orientation, clinical practice and diagnosis, and applications in healthcare. The final two questions were about perceived risks. Two items in this section, “AI will replace physicians in the future” and “AI would be a burden for practitioners,” were reversely scored to maintain consistency with the positively worded questions. The Cronbach’s alpha for the attitude section was 0.76, yielding satisfactory internal consistency.

#### Section IV – Practice.

The practice section consisted of 7 items to measure the frequency of AI use among medical students. These items measured the frequency of using AI for exam preparation, doing assignments, conducting research, idea generation, career guidance, personality development, and correction of grammatical and spelling errors. The practice section had a good Cronbach’s alpha, which was estimated to be 0.80.

#### Section V – Perceived barriers.

The final section of the study was conceptualized to illustrate the main barriers that hinder students’ usage of AI and included eight options, allowing students to select all that apply to them. These options include the following: lack of knowledge and expertise, ethical and privacy concerns, complexity of AI, lack of teaching centers, lack of access, lack of time due to educational burden, limited integration in educational curricula, and fear of over-reliance on AI.

#### Score categorization and interpretation.

Agreement and frequency 5-point Likert scales were used to assess responses to each statement in the KAP sections. The agreement 5-point Likert scale was used in the knowledge and attitude sections, with scores ranging from 1 denoting “strongly disagree” to 5 denoting “strongly agree”. The frequency 5-point Likert scale was used in the practice section, with scores ranging from 1 representing “never” to 5 representing “all the time”. The potential total score for the knowledge part ranged from 11 to 55, for the attitude section ranged from 12 to 60, while the practice section generated a potential score between 7 and 35. Participants’ scores in these sections were categorized according to Bloom’s criteria, in which a score above (80%) indicated a good/positive score, a score between (79%) and (60%) was termed as moderate/fair, and a score below (60%) was considered poor/negative. Higher scores reflected a higher level of knowledge, more positive attitudes, and better practices.

The use of the modified Bloom’s cutoff criteria for categorizing the KAP domains was based on several considerations. First, to our knowledge, there are no universally accepted or locally validated cutoff values for classifying KAP scores. Therefore, no single recommended cutoff exists. Second, given the increasing importance of AI in healthcare and medical education, we considered the use of a more stringent cutoff value to appropriately identify students with adequate AI preparedness and areas requiring improvement. Moreover, this cutoff criterion has been widely used in KAP studies, facilitating interpretation of the findings and comparison with previous KAP studies conducted across different populations and settings [16]. However, to avoid any potential concerns, a sensitivity analysis was performed using dependent variables as continuous measures.

### Inclusion and exclusion criteria

The inclusion criteria comprised medical students enrolled at Zakho Medical College and Duhok Medical College, aged 18 years or above, and who provided written or electronic consent to be recruited in the study. Conversely, pharmacy and dental students were excluded from the study. Furthermore, first-year medical students were also excluded because they started their educational year late and had limited exposure to medical school education. Finally, medical students who did not provide informed consent, as well as questionnaires with incomplete data or missing information, were excluded from the study.

### Ethical consideration

The study questionnaire was approved by the Ethics and Scientific Committee of the College of Medicine, University of Zakho, Kurdistan Region of Iraq on 15^th^ September, 2025 under the reference number (SEP2025/UOZEA060). Participation was entirely voluntary, and all participants provided written informed consent before recruitment. Additionally, data confidentiality and anonymity were strictly maintained, and data were exclusively utilized for research purposes. Ethical Standards reported in the Declaration of Helsinki for Medical Research Involving Human Subjects were followed.

### Statistical analysis

Microsoft Excel software and IBM SPSS version 30 were used for cleaning and analysis. The study dataset was cleaned and coded using Microsoft Excel, and the final coded dataset was transferred to IBM SPSS version 30 for statistical analysis. Descriptive statistics were summarized as frequencies and percentages. Whereas, continuous variables were represented as means and standard deviations. Crude odds ratios (cORs) and adjusted odds ratios (aORs) with (95%) confidence intervals (CIs) were utilized to establish statistical associations between participants’ demographic characteristics and KAP. All demographic characteristics were included in the adjusted logistic regression model. A p-value less than 0.05 was regarded as a statistically significant association. Additionally, sensitivity analysis using dependent variables as continuous measures was performed to ensure robustness and consistency of the results. Complete details and outcomes of the sensitivity analysis are available in S1A Table in S1 Word File.

### Declaration of generative AI

During the preparation of this manuscript, the authors used ChatGPT 4o in order to improve the grammar and linguistic format of the manuscript. After using this tool, the authors reviewed and edited the content as needed and take full responsibility for the content of the published article.

## Results

### Demographic characteristics

The mean age of the study participants was 21.31 ± 1.7 SD years, and the representation between both genders was approximately equal. Approximately half of the participants were in the pre-clinical stages. Out of all participants, about two-fifths (44.3%) had a cumulative academic average >70%. Only one-fifth of the students had attended workshops or seminars about the role of AI in healthcare and medical education. However, students from Zakho Medical College were significantly more likely to have attended such workshops or seminars than students from Duhok Medical College (25.5% vs. 16.6%; p = 0.015) (S2A Table). Moreover, about three-quarters of the participants reported that their college had not taken any steps toward integrating AI into education or training. Table 1 summarizes demographic characteristics of the study participants.

**Table 1:** Demographic characteristics of the participants (n = 575).

Variables	n (%)
**Age (Year)**	
21 and below	317 (55.1)
22 and above	258 (44.9)
Mean (SD)	21.31 (1.7)
**Gender**	
Male	294 (51.1)
Female	281 (48.9)
**College**	
Zakho Medical College	196 (34.1)
Duhok Medical College	379 (65.9)
**Stage**	
Pre-clinical	289 (50.3)
Clinical	286 (49.7)
**Cumulative academic average >70%**	
Yes	255 (44.3)
No	320 (55.7)
**Have you ever attended a workshop or seminar about role of AI in medical education and healthcare?**	
Yes	113 (19.7)
No	462 (80.3)
**Has your college taken any steps toward incorporating AI into education or training?**	
Yes	127 (22.1)
No	448 (77.9)

### Sources of information about AI

Fig 2 shows sources of information about AI among students. The internet/social media was the most common information source utilized by the students; this was followed by classmates and colleagues. Lastly, the college curriculum was the least informative source of information among medical students. Moreover, the sources of information about AI differed significantly between students from Zakho and Duhok Medical Colleges (S2A Table).

**Fig 2:**
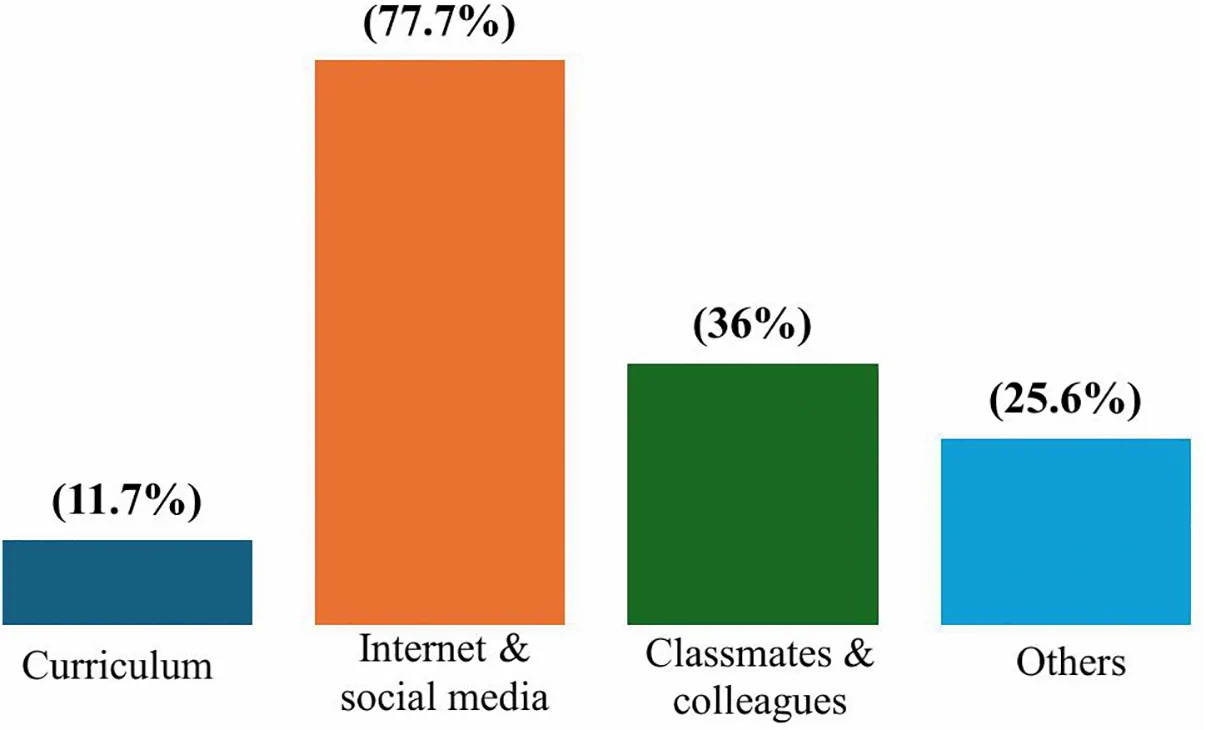
Sources of AI information among students.

### Students’ knowledge regarding AI

**S2B Table** demonstrates students’ knowledge about AI. About three-quarters (75.6%) of the students were aware of AI, and about half (45.2%) of the participants reported having solid knowledge about the basics of Al. Furthermore, about two-fifths were knowledgeable about subtypes of AI, including machine learning and deep learning. About three-quarters (71.6%) agreed that AI may make errors when trained on biased or incomplete data, and about two-thirds strongly agreed or agreed that AI needs large amounts of labeled data to learn. Additionally, about half of the students knew that AI applications can be used in radiology, pathology, and cardiology.

### Attitudes toward AI

About three-fifths of the students either strongly agreed or agreed that AI has a positive impact on students’ performance. Moreover, about half of participants strongly agreed or agreed that AI will revolutionize education and could be used as a tool for assessment. Nearly, half of the respondents reported that AI had affected their future career orientation. Similarly, (47.7%) reported that AI will aid practitioners in assessing the severity of the disease, and about two-fifths said that AI will decrease the percentage of medical errors. Only, (6.8%) strongly agreed that physicians will be replaced by AI in the future. Fig 3 provides details of participants’ attitudes toward AI.

**Fig 3:**
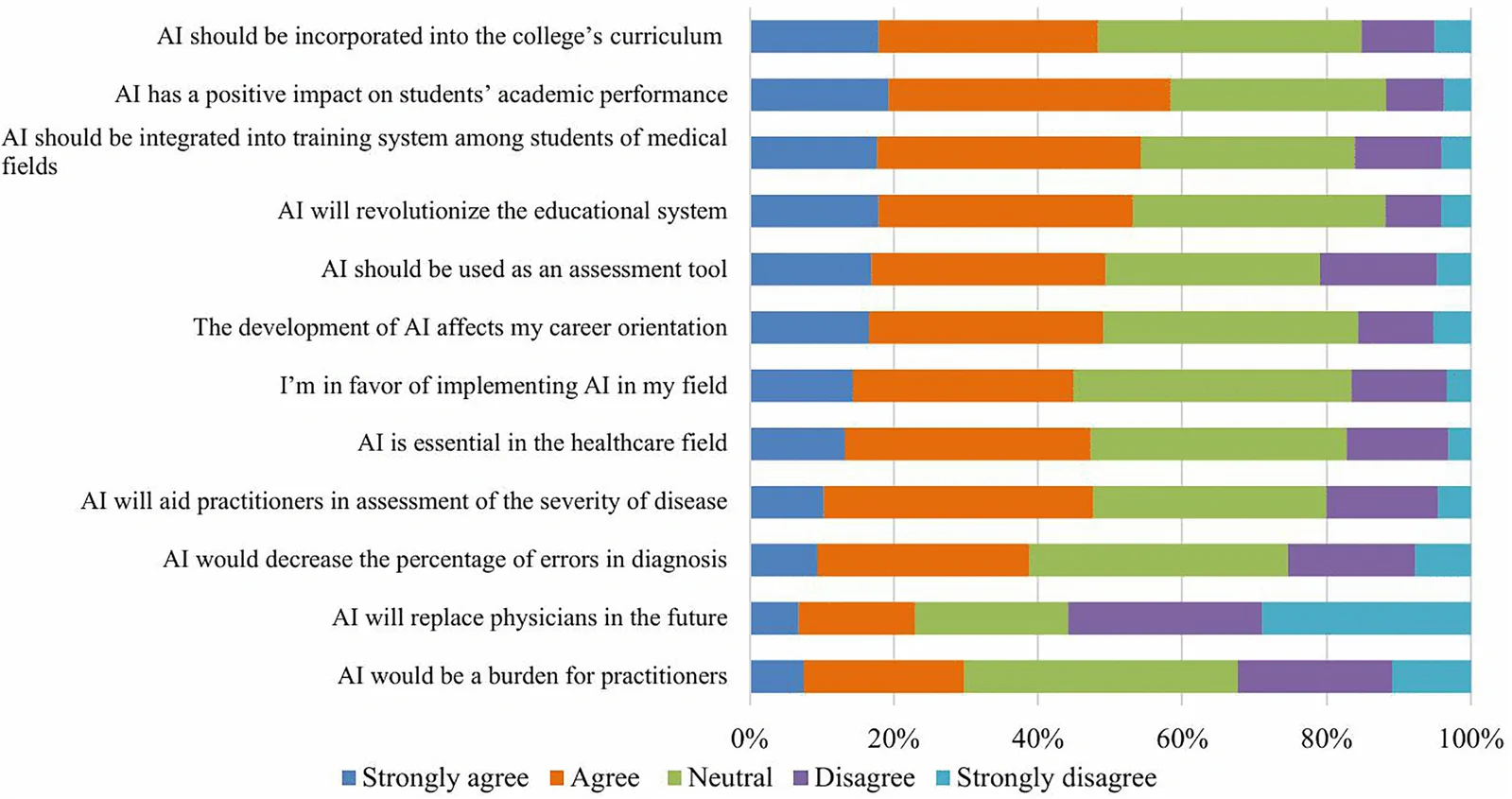
Participants’ attitudes toward AI.

### Participants’ practices regarding AI

S2C Table illustrates respondents’ practices toward AI. Among the students, AI was most frequently used for homework/assignments (56.5%) and exam preparation (55.8%). Additionally, about two-fifths of the participants reported using AI for research and brainstorming. AI was less frequently used for personal choices and career guidance. Moreover, about one-fifth of the students rarely used AI for spelling and grammar checking.

### Perceived barriers hindering the use of AI

The most significant barrier limiting the use of AI among students was fear of over-reliance (53.2%). This was followed by lack of time due to educational burden, which was reported by around two-fifths. Lack of knowledge and expertise and lack of teaching centers were also prominent barriers. Fig 4 demonstrates the complete list of barriers hindering the use of AI.

**Fig 4:**
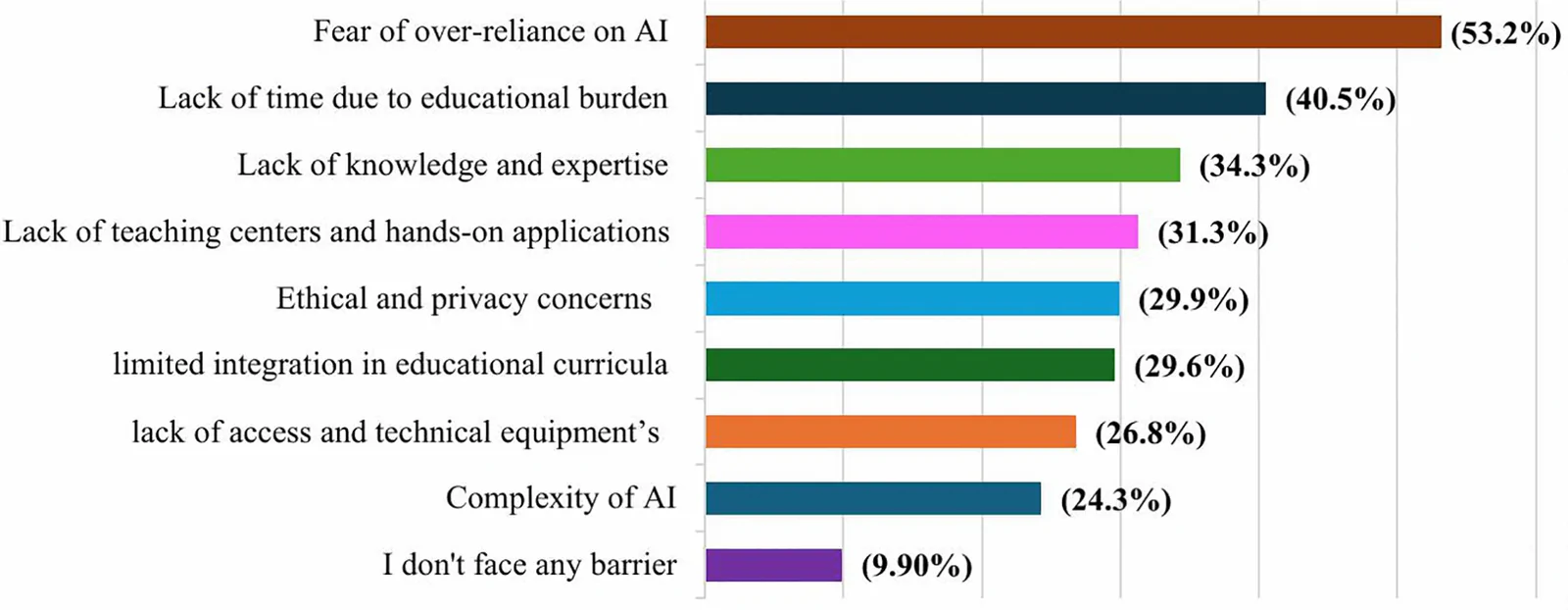
Barriers hindering the use of AI.

### Classification of knowledge, attitudes, and practices levels

Table 2 demonstrates the classification of participants’ KAP according to the modified Bloom’s cut-off criteria. Approximately one-quarter of the participants demonstrated good knowledge of AI, whereas only (13.9%) of students exhibited a positive attitude toward AI. In contrast, the practice domain had the highest proportion of participants classified as having poor AI-related practices.

**Table 2:** Knowledge, attitude, and practice classification (n = 575).

Variables	Good n (%)	Moderate n (%)	Poor n (%)
Knowledge	132 (23)	352 (61.2)	91 (15.8)
Attitudes	80 (13.9)	404 (70.3)	91 (15.8)
Practice	88 (15.3)	270 (47)	217 (37.7)

### Determinants of good AI knowledge

Gender was the only statistically significant factor associated with good AI knowledge. Male students demonstrated higher odds of good knowledge in both unadjusted and adjusted models. On the contrary, other demographic factors such as age, college, stage, cumulative academic average, attending workshops, and source of information did not show a significant statistical association. Table 3 presents univariate and multivariate analysis of association between demographic factors and good AI knowledge.

**Table 3:** Determinants of good AI knowledge among students (n = 575).

Variables	Good n (%) 132 (23.0)	Moderate/Poor n (%) 443 (77.0)	Crude OR [95% CI; p-value]	Adjusted OR [95% CI; p-value]
**Age (Year)**				
21 and below	74 (23.3)	243 (76.7)	Ref	Ref
22 and above	58 (22.5)	200 (77.5)	0.95 [0.64–1.41; p = 0.81]	0.89 [0.52–1.51; p = 0.67]
**Gender**				
Male	93 (31.6)	201 (68.4)	Ref	Ref
Female	39 (13.9)	242 (86.1)	0.35 [0.23–0.53; p < 0.001]	0.34 [0.22–0.51; p < 0.001]
**College**				
Zakho Medical College	48 (24.5)	148 (75.5)	Ref	Ref
Duhok Medical College	84 (22.2)	295 (77.8)	0.88 [0.59–1.32; p = 0.53]	0.83 [0.53–1.30; p = 0.41]
**Stage**				
Pre-clinical	67 (23.2)	222 (76.8)	Ref	Ref
Clinical	65 (22.7)	221 (77.3)	0.98 [0.66–1.44; p = 0.90]	1.14 [0.66–1.97; p = 0.64]
**Cumulative academic average >70%**				
Yes	63 (24.7)	192 (75.3)	Ref	Ref
No	69 (21.6)	251 (78.4)	0.84 [0.57–1.24; p = 0.37]	0.82 [0.55–1.24; p = 0.36]
**Attended a workshop or seminar about AI**				
Yes	28 (24.8)	85 (75.2)	Ref	Ref
No	104 (22.5)	358 (77.5)	0.88 [0.55–1.43; p = 0.61]	0.96 [0.57–1.61; p = 0.88]
**Has your college incorporated AI into education**				
Yes	36 (28.4)	91 (71.6)	Ref	Ref
No	96 (21.4)	352 (78.6)	0.69 [0.44–1.08; p = 0.10]	0.67 [0.41–1.08; p = 0.09]
**Sources of information about AI**				
Classmates & Colleagues	7 (17.5)	33 (82.5)	Ref	Ref
College curriculum	3 (37.5)	5 (62.5)	2.83 [0.55–14.69; p = 0.22]	2.36 [0.42–13.18; p = 0.33]
Internet/social media	50 (20.7)	192 (79.3)	1.23 [0.51–2.94; p = 0.65]	1.09 [0.44–2.68; p = 0.85]
Others	16 (23.9)	51 (76.1)	1.48 [0.55–3.90; p = 0.44]	1.29 [0.46–3.60; p = 0.63]
Combination of ≥ 2 sources	56 (25.7)	162 (74.3)	1.63 [0.68–3.89; p = 0.27]	1.55 [0.64–3.80; p = 0.34]

### Predictors of positive AI attitudes

Table 4 illustrates factors associated with positive attitudes using binary logistic regression. Gender and college affiliation were significant predictors of positive AI attitudes among students. Female students had lower odds (cOR: 0.45; 95% CI: 0.28–0.75; p = 0.002) of a positive attitude towards AI compared with male students. The finding remained statistically significant and showed a similar trend even after adjustment for confounders (aOR: 0.46; 95% CI: 0.28–0.77; p = 0.003). Furthermore, students from Duhok Medical College were less likely to have a positive attitude toward AI in both regression models. However, this variable did not reach statistical significance in the sensitivity analysis model (S1A Table in S1 Word File).

**Table 4:** Factors associated with a positive attitude toward AI among participants (n = 575).

Variables	Positive n (%) 80 (13.9)	Fair/Negative n (%) 495 (86.1)	Crude OR [95% CI; p-value]	Adjusted OR [95% CI; p-value]
**Age (Year)**				
21 and below	43 (13.6)	274 (86.4)	Ref	Ref
22 and above	37 (14.3)	221 (85.7)	1.07 [0.66–1.71; p = 0.79]	0.95 [0.51–1.79; p = 0.88]
**Gender**				
Male	54 (18.4)	240 (81.6)	Ref	Ref
Female	26 (9.3)	255 (90.7)	0.45 [0.28–0.75; p = 0.002]	0.46 [0.28–0.77; p = 0.003]
**College**				
Zakho Medical College	36 (18.4)	160 (81.6)	Ref	Ref
Duhok Medical College	44 (11.6)	335 (88.4)	0.58 [0.36–0.94; p = 0.028]	0.52 [0.30–0.88; p = 0.014]
**Stage**				
Pre-clinical	40 (13.8)	249 (86.2)	Ref	Ref
Clinical	40 (14)	246 (86)	1.01 [0.63–1.62; p = 0.96]	1.19 [0.62–2.31; p = 0.60]
**Cumulative academic average >70%**				
Yes	35 (13.7)	220 (86.3)	Ref	Ref
No	45 (14.1)	275 (85.9)	1.03 [0.64–1.66; p = 0.91]	0.91 [0.56–1.50; p = 0.72]
**Attended a workshop or seminar about AI**				
Yes	12 (10.6)	101 (89.4)	Ref	Ref
No	68 (14.7)	394 (85.3)	1.45 [0.76–2.79; p = 0.26]	1.42 [0.72–2.83; p = 0.31]
**Has your college incorporated AI into education**				
Yes	13 (10.2)	114 (89.8)	Ref	Ref
No	67 (15)	381 (85)	1.54 [0.82–2.90; p = 0.18]	1.49 [0.77–2.87; p = 0.24]
**Sources of information about AI**				
Classmates & Colleagues	4 (10)	36 (90)	Ref	Ref
College curriculum	2 (25)	6 (75)	3 [0.45–20.15; p = 0.26]	3.23 [0.45–23.79; p = 0.24]
Internet/social media	36 (14.9)	206 (85.1)	1.57 [0.53–4.69; p = 0.42]	1.38 [0.45–4.21; p = 0.57]
Others	8 (11.9)	59 (88.1)	1.22 [0.34–4.35; p = 0.76]	1.22 [0.33–4.48; p = 0.76]
Combination of ≥ 2 sources	30 (13.8)	188 (86.2)	1.44 [0.48–4.33; p = 0.52]	1.36 [0.44–4.21; p = 0.59]

### Predictor of better AI-related practices

Students who relied on other sources of information had significantly lower odds of good AI-related practices compared with those who relied on classmates and colleagues (cOR: 0.09; 95% CI: 0.02–0.45; p = 0.003). This association remained statistically significant in the multivariable model (aOR: 0.09; 95% CI: 0.02–0.45; p = 0.003). No statistically significant associations were observed for other variables in either unadjusted or adjusted models. Table 5 presents the predictors of practices among students. Notably, in the sensitivity analysis model (S1A Table in S1 Word File), students from Zakho Medical College significantly exhibited better AI practices, and the direction of association was consistent across both the primary and sensitivity analysis models.

**Table 5:** Predictors of better AI-related practices among students (n = 575).

Variables	Good n (%) 88 (15.3)	Moderate/Poor n (%) 487 (84.7)	Crude OR [95% CI; p-value]	Adjusted OR [95% CI; p-value]
**Age (Year)**				
21 and below	54 (17)	263 (83)	Ref	Ref
22 and above	34 (13.2)	224 (86.8)	0.74 [0.47–1.18; p = 0.20]	0.89 [0.48–1.63; p = 0.70]
**Gender**				
Male	46 (15.6)	248 (84.4)	Ref	Ref
Female	42 (14.9)	239 (85.1)	0.95 [0.60–1.49; p = 0.82]	0.90 [0.56–1.43; p = 0.65]
**College**				
Zakho Medical College	36 (18.4)	160 (81.6)	Ref	Ref
Duhok Medical College	52 (13.7)	327 (86.3)	0.71 [0.44–1.13; p = 0.14]	0.80 [0.48–1.33; p = 0.39]
**Stage**				
Pre-clinical	51 (17.6)	238 (82.4)	Ref	Ref
Clinical	37 (12.9)	249 (87.1)	0.69 [0.44–1.01; p = 0.12]	0.83 [0.44–1.55; p = 0.55]
**Cumulative academic average >70%**				
Yes	41 (16.1)	214 (83.9)	Ref	Ref
No	47 (14.7)	273 (85.3)	0.90 [0.57–1.42; p = 0.65]	0.91 [0.57–1.46; p = 0.70]
**Attended a workshop or seminar about AI**				
Yes	22 (19.5)	91 (80.5)	Ref	Ref
No	66 (14.3)	396 (85.7)	0.69 [0.40–1.18; p = 0.17]	0.72 [0.41–1.23; p = 0.24]
**Has your college incorporated AI into education**				
Yes	21 (16.5)	106 (83.5)	Ref	Ref
No	67 (15)	381 (85)	0.89 [0.52–1.52; p = 0.66]	0.96 [0.55–1.69; p = 0.90]
**Sources of information about AI**				
Classmates & Colleagues	10 (25)	30 (75)	Ref	Ref
College curriculum	2 (25)	6 (75)	1 [0.17–5.77; p = 1]	1.10 [0.18–6.57; p = 0.92]
Internet/social media	39 (16.1)	203 (83.9)	0.58 [0.26–1.27; p = 0.17]	0.55 [0.24–1.22; p = 0.14]
Others	2 (3)	65 (97)	0.09 [0.02–0.45; p = 0.003]	0.09 [0.02–0.45; p = 0.003]
Combination of ≥ 2 sources	35 (16.1)	183 (83.9)	0.57 [0.26–1.28; p = 0.17]	0.58 [0.26–1.29; p = 0.18]

## Discussion

The rapid advancement of AI in recent years has led to its inevitable integration into both healthcare and medical education. A systematic review has reported that despite increasing awareness of AI among health professions students, many still possess limited knowledge and lack the technical skills required to use AI effectively in medical settings [17]. Data exploring students’ KAP regarding AI in the Kurdistan Region of Iraq remain scarce. Therefore, this study aimed to address this literature gap. Overall, the study found that the majority of medical students have not attended any workshops related to the role of AI in healthcare and medical education. Additionally, AI appears to be poorly integrated into current college curricula. Unfortunately, in terms of students’ KAP, suboptimal results were obtained, with the practice domain having the highest proportion of participants classified as having poor AI-related practices.

The present study identified a significant gap in both teaching and institutional integration of AI. The majority of students reported that they had not attended any workshops on AI and that their college had not taken any steps to incorporate AI into the curriculum. Similarly, our findings are in line with those of a multinational study conducted among Arab medical students, which reported that (92.4%) of their students had not received formal training in AI [16]. The absence of structured educational programs may lead students to rely on unofficial and non-academic sources of information. In our study, internet/social media appeared as a primary source of AI-related information, a finding consistent with a study conducted in Cyprus [18]. The lack of formal AI training, combined with reliance on less reliable resources, may result in students acquiring only superficial knowledge rather than a deep understanding of AI, thereby limiting their ability to adequately utilize and advance it.

In terms of students’ knowledge, the overall knowledge level was unsatisfactory. Most students were aware of the basic concepts of AI. However, their knowledge significantly declined when exploring AI subtypes, such as machine learning and deep learning, as well as its applications across different medical specialties. Our findings are consistent with regional studies conducted in Syria and Lebanon, where students also had a good understanding of AI basics, but demonstrated partial awareness about core concepts and AI applications in medicine [3,15]. The limited depth of understanding regarding AI subtypes and their applications among medical students may be attributed to the lack of formal teaching initiatives.

Generally, the study participants exhibited a positive attitude toward the incorporation of AI into healthcare and medical education. Approximately half of the respondents expressed a favorable attitude toward integrating AI into medical curricula. Another study carried out in Iraq reported even more positive attitudes among their students, where the vast majority supported the inclusion of AI in their field of study [19]. Consistently, about four-fifths of undergraduate medical students in India believed that AI should be part of medical training systems [20]. Furthermore, nearly half of the students indicated that AI had influenced their career orientation. This finding is not unique to our study alone; a study from Sweden reported that about one-third of medical students intended to avoid specialties perceived to be threatened by AI, particularly radiology and pathology [21]. These findings highlight the importance of educating students to understand the role of AI as a tool that can advance medical practice, rather than as a threat to the specialties.

Taken together, our findings and those reported in previous studies suggest that students generally favor integration of AI into medical education. However, several ethical challenges and concerns arise when considering the integration of AI and LLMs into medical education, of which students may not be fully aware. These challenges include issues related to data security and privacy, and the lack of transparency in AI decisions may result in distrust regarding the accuracy of its outputs. Moreover, hallucinations generated by AI and LLMs may provide false information, generate data in the absence of sufficient information, or even produce fabricated references due to their inability to provide a specific source. Additionally, the inability of these algorithms to account for cultural diversity may lead to unfair and biased education for certain groups of people. Responsibility and accountability are also among the most prominent ethical concerns related to AI integration [11]. Therefore, these challenges and concerns should not be overlooked when planning the integration of AI into medical education.

The current study demonstrated that a considerable proportion of students have started to frequently utilize AI in their daily tasks. It was evident that these tools were primarily used for academic purposes, most commonly for homework/assignments and preparation for exams. Our results are consistent with those of studies carried out among medical students in Uganda and health sciences students in Palestine, which reported that AI was most frequently utilized for completing academic assignments [22,23]. However, our findings differ from those of a Jordanian study carried out among health professions students, where a smaller proportion reported using AI for their academic tasks, and the most frequent reason for using AI was for spelling and grammar checking [13].

This difference in AI utilization between both studies could be explained by the timing of the Jordanian study, which was conducted in 2023, when AI tools were relatively recent, and students may have had limited awareness about their availability and usage. We recommend that students should not be discouraged from using these valuable tools. However, they should be guided and taught how to use these tools appropriately and reasonably. Additionally, when seeking medical information, they should only depend on high-quality tools that provide accurate, evidence-based content, and the generated content should be manually checked by users.

Despite this relatively common use of AI tools, several barriers hindering effective utilization of AI were identified. The most commonly reported barrier included fear of over-reliance on AI tools. This is an optimistic finding, which suggests that students are aware of potential risks associated with AI and that excessive dependence on AI tools could compromise their critical thinking and learning skills. Moreover, a study conducted among doctors and medical students in Saudi Arabia found that lack of proper training was the primary reason for underutilization of AI [24]. Additionally, ethical concerns, issues related to data accuracy, and limited AI literacy were reported as major challenges among Palestinian medical students [25].

In the present study, female participants had significantly lower odds of demonstrating good knowledge and a positive attitudes toward AI compared with male participants. Our findings are in line with studies conducted among Egyptian and Indonesian medical students, which also reported that male students had significantly better knowledge of AI than their female counterparts [26,27]. Moreover, a study carried out among health sciences students in Northern Saudi Arabia found no significant association between gender and AI knowledge. However, contrary to our finding, that study reported that female students significantly exhibited more positive attitudes compared to males [28].

The observed gender difference could be attributed to the fact that male students have greater interest and exposure to technology, computers, and digital tools. In the absence of formal AI integration into the curriculum, prior experiences and exposure may contribute to disparity in AI usage. Female students might have limited opportunities and engagement in AI. Therefore, it’s important to ensure equitable AI training and opportunities for both genders. In support with our findings, a systematic review conducted among higher education students reported that male students tended to use AI tools more broadly, whereas female students generally adopted AI tools more cautiously, with particular attention to ethical concerns, transparency, and the need for structured guidance. The review further highlighted that cultural and social norms affect students’ engagement with AI [29].

Moreover, students from Duhok Medical College had lower odds of demonstrating a positive attitudes toward AI compared to students from Zakho Medical College. The data from the present study showed that students from Zakho Medical College had greater attendance at workshops or seminars on the role of AI in healthcare and medical education, and this difference was statistically significant. Additionally, there were significant differences between sources of AI information reported by students from these two institutions. These differences in students’ educational experiences and information sources may have partly contributed to the observed variation in attitudes toward AI between both medical colleges.

### Strengths and limitations

This study is strengthened by being among the few studies exploring medical students’ perceptions of AI in the Kurdistan Region of Iraq, and by performing sensitivity analysis to ensure robustness and consistency of the findings. Nevertheless, several limitations should be acknowledged. First, the convenience sampling method may have resulted in selection bias. Additionally, the potential for self-report bias and social desirability bias exists. Third, the cross-sectional nature of the study design cannot infer causal relationships. In addition, data were collected from only two medical colleges. Therefore, the findings cannot be generalized to all medical students in the Kurdistan Region of Iraq. However, we believe that this study provides a valuable landscape for future research on AI among medical students in the Kurdistan Region of Iraq.

## Conclusions

The current study exhibited suboptimal levels of KAP regarding AI among medical students. The study found that AI integration into medical curriculum remains limited in our region. Furthermore, the internet/social media was identified as the primary source of information about AI, and students reported using AI most frequently for homework/assignments. The most common barrier for AI utilization was fear of over-reliance. Additionally, male students demonstrated higher levels of knowledge, and more positive attitudes toward AI compared to female students.

### Recommendations

AI is becoming an integral component of healthcare and medical education, with the potential to substantially advance both fields. Globally, many high-income countries have taken significant steps toward the widespread adaptation and utilization of this technology. However, such integration remains less evident in LMICs. Therefore, several measures should be considered by our universities and colleges. Medical colleges in the Kurdistan Region of Iraq may consider integrating AI into practical training, which may enhance students’ educational experiences and support students in practicing complex decision-making in an environment that simulates real-world practice. Furthermore, medical institutions should educate their students with both foundational and advanced AI concepts in order to enhance students’ understanding of potential concerns and biases associated with AI technologies, and to guide them toward optimal and responsible use. Finally, future multicenter studies are needed to better understand the benefits and potential harms of AI in healthcare and medical education, while the development of validated AI literacy tools may also be valuable.

## Supporting information

S1 Word File. Sensitivity analysis. Contains Table 1A.
(DOCX)

S2 Tables Institutional differences in AI exposure and knowledge and attitudes of students toward AI. Contains Table 2A, Table 2B, and Table 2C.
(DOCX)

S3 Data Minimal dataset. (XLSX)
