# Supplementary material for: Medical students’ knowledge, attitudes, and practices regarding artificial intelligence: A cross-sectional study
Source: PLoS One. 2026 Oct 27;21(10):e0350001. doi: 10.1371/journal.pone.0350001 (PMC13712711; doi:10.1371/journal.pone.0350001)
Supplement: Supplementary file 1 [file pone.0350001.s001.docx]

**Supplementary Material 1**

**Sensitivity analysis**

Sensitivity analysis was executed to evaluate robustness and consistency of the findings. In this study, we performed sensitivity analysis modeling dependent variables as continuous measures to avoid any statistical association generated due to specific cutoff, and to increase statistical power. The data were tested for normality using Shapiro-Wilk test, and the test revealed non-normal distribution of the data; hence, non-parametric tests were employed to calculate statistical association. When comparing two independent groups the two-tailed Mann-Whitney U test was utilized, whereas the Kruskal-Wallis H test was used for comparisons between more than two independent groups. **Table A** shows complete results of sensitivity analysis using continuous score modeling.

| **Table 1A. Sensitivity analysis of determinants of knowledge, attitudes, and practices using continuous dependent variables model (n=575)** | | | | | | |
| --- | --- | --- | --- | --- | --- | --- |
| **Variables** | **Knowledge**  **Median [IQR]** | **p-value** | **Attitude**  **Median [IQR]** | **p-value** | **Practice**  **Median [IQR]** | **p-value** |
| **Age (Year)** |  |  |  |  |  |  |
| 21 and below | 39 [36-43] | 0.077 | 40 [37-45] | 0.21 | 22 [19-25] | 0.39 |
| 22 and above | 38.5 [34-43] |  | 42 [37-45] |  | 22 [18-25] |  |
| **Gender** |  |  |  |  |  |  |
| Male | 40 [35-45] | <0.001 | 42 [37-46] | 0.002 | 22 [18-25] | 0.21 |
| Female | 38 [35-41] |  | 40 [37-43] |  | 22 [19-26] |  |
| **College** |  |  |  |  |  |  |
| Zakho Medical College | 38 [35-43] | 0.93 | 41 [37-45] | 0.40 | 23 [20-26] | 0.005 |
| Duhok Medical College | 39 [35-43] |  | 41 [37-44] |  | 21 [18-25] |  |
| **Stage** |  |  |  |  |  |  |
| Pre-clinical | 39 [35-43] | 0.22 | 41 [37-45] | 0.67 | 22 [19-25.5] | 0.08 |
| Clinical | 39 [34-43] |  | 42 [37-45] |  | 22 [18-25] |  |
| **Cumulative academic average >70%** |  |  |  |  |  |  |
| Yes | 39 [35-43] | 0.054 | 41 [37-44] | 0.91 | 22 [18-25] | 0.11 |
| No | 38 [34.25-43] |  | 41 [37-45] |  | 22 [19-26] |  |
| **Attended a workshop or seminar about AI** |  |  |  |  |  |  |
| Yes | 39 [35-43.5] | 0.61 | 41 [37-44] | 0.66 | 22 [19-26] | 0.24 |
| No | 39 [35-43] |  | 41 [37-45] |  | 22 [18-25] |  |
| **Has your college incorporated AI into education** |  |  |  |  |  |  |
| Yes | 39 [35-44] | 0.39 | 41 [37-44] | 0.32 | 22 [19-26] | 0.76 |
| No | 39 [35-43] |  | 41 [37-45] |  | 22 [18-25] |  |
| **Sources of information about AI** |  |  |  |  |  |  |
| Classmates & Colleagues | 39.5 [36.25-42] | 0.54 | 40 [37.25-43] | 0.26 | 21.5 [19-27.5] | 0.06 |
| College curriculum | 41 [36-46.25] |  | 43 [36.25-49.25] |  | 22.5 [19.75-27] |  |
| Internet/social media | 38.5 [35-43] |  | 41 [37-44.25] |  | 22 [19-26] |  |
| Others | 39 [32-43] |  | 41 [35-44] |  | 21 [18-23] |  |
| Combination ≥ 2 sources | 39 [35-44] |  | 42 [38-45] |  | 22 [18-25] |  |
