# Supplementary material for: Medical students’ knowledge, attitudes, and practices regarding artificial intelligence: A cross-sectional study
Source: PLoS One. 2026 Oct 27;21(10):e0350001. doi: 10.1371/journal.pone.0350001 (PMC13712711; doi:10.1371/journal.pone.0350001)
Supplement: Supplementary file 2 [file pone.0350001.s002.docx]

**Supplementary Material 2**

| **Table 2A. Institutional differences in students’ AI exposure and information sources (n=575)** | | | |
| --- | --- | --- | --- |
| **Variables** | **Zakho Medical College n (%)** | **Duhok Medical College n (%)** | **p-value** |
| **Have you ever attended a workshop or seminar about role of AI in medical education and healthcare?** |  |  |  |
| Yes | 50 (25.5) | 63 (16.6) | 0.015^*^ |
| No | 146 (74.5) | 316 (83.4) |  |
| **Has your college taken any steps toward incorporating AI into education or training?** |  |  |  |
| Yes | 52 (26.5) | 75 (19.7) | 0.072^*^ |
| No | 144 (73.5) | 304 (80.2 |  |
| **Sources of information about AI** |  |  |  |
| Classmates & Colleagues | 12 (6.1) | 28 (7.4) | <0.001^#^ |
| College Curriculum | 1 (0.5) | 7 (1.8) |  |
| Internet/social media | 106 (54.1) | 136 (35.9) |  |
| Others | 15 (7.7) | 52 (13.7) |  |
| Combination of ≥ 2 sources | 62 (31.6) | 156 (41.2) |  |
| *Fischer’s exact test, ^#^Pearson chi-square | | | |

| **Table 2B. Students’ knowledge about AI (n=575)** | | | | | |
| --- | --- | --- | --- | --- | --- |
| **Variables** | **Strongly agree n (%)** | **Agree**  **n (%)** | **Neutral**  **n (%)** | **Disagree**  **n (%)** | **Strongly disagree n (%)** |
| 1. I know what AI is | 216 (37.5) | 219 (38.1) | 89 (15.5) | 27 (4.7) | 24 (4.2) |
| 2. I have solid knowledge of the basics of AI | 104 (18.1) | 156 (27.1) | 223 (38.8) | 72 (12.5) | 20 (3.5) |
| 3. I know that machine learning is a subtype of AI | 70 (12.2) | 146 (25.4) | 218 (37.9) | 104 (18.1) | 37 (6.4) |
| 4. I know that deep learning is a subtype of AI | 73 (12.7) | 153 (26.6) | 207 (36) | 107 (18.6) | 35 (6.1) |
| 5. AI models may make errors if trained on biased or incomplete data | 217 (37.7) | 195 (33.9) | 106 (18.5) | 38 (6.6) | 19 (3.3) |
| 6. AI may make errors and requires human oversight | 129 (22.4) | 166 (28.9) | 102 (17.7) | 100 (17.4) | 78 (13.6) |
| 7. AI requires a lot of labeled data to learn (data already processed by humans) | 190 (33) | 195 (33.9) | 120 (21) | 48 (8.3) | 22 (3.8) |
| 8. I am aware of applications of AI in my field of interest. | 100 (17.4) | 225 (39.1) | 160 (27.8) | 67 (11.7) | 23 (4) |
| 9. AI can be applied in radiology and detect abnormalities in medical images | 109 (18.9) | 197 (34.3) | 148 (25.7) | 82 (14.3) | 39 (6.8) |
| 10. AI can be applied in pathology and analyze slides to detect diseases | 85 (14.9) | 198 (34.4) | 172 (29.9) | 79 (13.7) | 41 (7.1) |
| 11. AI can be applied in cardiology (Example: ECG analysis) | 89 (15.5) | 182 (31.7) | 168 (29.2) | 84 (14.6) | 52 (9) |

| **Table 2C. Medical students’ practices toward AI (n=575)** | | | | | |
| --- | --- | --- | --- | --- | --- |
| **Variables** | **Never**  **n (%)** | **Rarely**  **n (%)** | **Often**  **n (%)** | **Most of the time**  **n (%)** | **All the time**  **n (%)** |
| 1. How frequently do you use AI to prepare for your exams? | 22 (3.8) | 44 (7.7) | 188 (32.7) | 196 (34.1) | 125 (21.7) |
| 2. How frequently do you use AI to prepare for your homework/assignments? | 11 (2) | 49 (8.5) | 190 (33) | 207 (36) | 118 (20.5) |
| 3. How frequently do you use AI to conduct your research? | 33 (5.7) | 86 (15) | 208 (36.2) | 154 (26.8) | 94 (16.3) |
| 4. How frequently do you use AI for idea  generation and brainstorming? | 22 (3.8) | 106 (18.4) | 207 (36.1) | 167 (29) | 73 (12.7) |
| 5. How frequently do you use AI for personal choices/career guidance? | 101 (17.6) | 164 (28.5) | 159 (27.6) | 100 (17.4) | 51 (8.9) |
| 6. How frequently do you use AI for spelling and grammar checking? | 91 (15.8) | 134 (23.3) | 185 (32.2) | 98 (17) | 67 (11.7) |
| 7. How frequently do you use AI for personality development and other skills? | 101 (17.6) | 145 (25.2) | 178 (30.9) | 101 (17.6) | 50 (8.7) |
